# Clinical Implications of Combinatorial Pharmacogenomic Tests Based on Cytochrome P450 Variant Selection

**DOI:** 10.3389/fgene.2021.719671

**Published:** 2021-09-28

**Authors:** Michael Sayer, Ashley Duche, Trang Jenny Tran Nguyen, Michelle Le, Kunj Patel, Jacqueline Vu, Danny Pham, Brianne Vernick, Richard Beuttler, Don Roosan, Moom R. Roosan

**Affiliations:** ^1^Department of Pharmacy Practice, Chapman University School of Pharmacy, Irvine, CA, United States; ^2^College of Pharmacy, Western University of Health Sciences, Pomona, CA, United States

**Keywords:** pharmacogenomics, pharmacogenomic tests, variant selection, detection rate, cytochrome P450 enzymes

## Abstract

Despite the potential to improve patient outcomes, the application of pharmacogenomics (PGx) is yet to be routine. A growing number of PGx implementers are leaning toward using combinatorial PGx (CPGx) tests (i.e., multigene tests) that are reusable over patients’ lifetimes. However, selecting a single best available CPGx test is challenging owing to many patient- and population-specific factors, including variant frequency differences across ethnic groups. The primary objective of this study was to evaluate the detection rate of currently available CPGx tests based on the cytochrome P450 (CYP) gene variants they target. The detection rate was defined as the percentage of a given population with an “altered metabolizer” genotype predicted phenotype, where a CPGx test targeted both gene variants a prospective diplotypes. A potential genotype predicted phenotype was considered an altered metabolizer when it resulted in medication therapy modification based on Clinical Pharmacogenetics Implementation Consortium (CPIC) guidelines. Targeted variant CPGx tests found in the Genetic Testing Registry (GTR), gene selection information, and diplotype frequency data from the Pharmacogenomics Knowledge Base (PharmGKB) were used to determine the detection rate of each CPGx test. Our results indicated that the detection rate of CPGx tests covering CYP2C19, CYP2C9, CYP2D6, and CYP2B6 show significant variation across ethnic groups. Specifically, the Sub-Saharan Africans have 63.9% and 77.9% average detection rates for CYP2B6 and CYP2C19 assays analyzed, respectively. In addition, East Asians (EAs) have an average detection rate of 55.1% for CYP2C9 assays. Therefore, the patient’s ethnic background should be carefully considered in selecting CPGx tests.

## Introduction

Drug-related morbidity and mortality owing to unoptimized medication therapy are estimated to cost $528.4 billion annually in the United States alone (Watanabe et al., 2018). Pharmacogenomics (PGx) – the study of the role of an individual’s genetic makeup in drug response – has the potential to reduce adverse reactions to medications and lower medical costs by individualizing treatments based on genetic makeup. In a study of Medicare and Medicaid patients examining over 70 million patient records, over half of the patient population received at least one drug with PGx implications (Samwald et al., 2016). Studies comparing PGx guided therapies vs. non-PGx guided therapies in psychiatric patients show both improved therapy outcomes and significant cost savings (Hornberger et al., 2015; Tanner et al., 2019). Clinical PGx practice has the potential to be relevant to a large patient population, bring cost savings, and improve therapy outcomes.

Critical to PGx implementation is the availability of genetic test results having relevant genes to guide therapy decisions. Reactionary approaches to PGx practice, meaning individual genes are tested when there is a suspected need, is inefficient with respect to cost and time. As clinical decision support systems grow, preemptive PGx testing approaches are being utilized, allowing genotyping results to be available prior to prescribing decisions and in the planning of therapy (Dunnenberger et al., 2015). Combinatorial PGx tests (CPGx) are critical to the development of these programs because they offer genotyping results of several different genes simultaneously. While utilizing CPGx tests preemptively helps overcome barriers to PGx implementation, it is important to ensure the genotyping results they provide are reliable. If CPGx tests utilized preemptively have limitations, they can cause sub-optimal outcomes for subsequent therapeutic decisions.

Cytochrome P450 (CYP) enzyme genotypes are relevant to PGx practice for a variety of reasons. CYP enzymes play a role in the metabolism of over 90% of available prescription medications (Lynch and Price, 2007; McDonnell and Dang, 2013). In addition to their critical role in drug metabolism, the prevalence of genetic polymorphisms of CYP enzymes is well documented in diverse patient populations. In a study of nearly 10,000 patients screened for common CYP enzyme variants, 91% of them had at least one variant linked to changed metabolic status (Van Driest et al., 2014). CYP Enzyme genotyping plays a central role in PGx practice for these reasons. Given this, the success of a preemptive PGx testing program with CPGx tests can be greatly influenced by the extent CYP enzyme genotypes are accurately characterized.

Gene variant selection of CPGx tests is an essential factor to consider that can influence therapeutic decisions (Petry et al., 2021). CPGx test performance can vary because laboratories providing PGx tests use targeted genotyping technologies to screen for specific variants with well-characterized drug-gene interactions (Guo et al., 2019). Currently, all target variant CPGx tests that do not find putative variants included in the test report the gene as a normal (*1) variant by default (Mukerjee et al., 2018). In a study of CYP2C9 genotype-guided warfarin dosing vs. standard clinical dosing in 2013, African American patients were in the therapeutic range significantly less with genotype-guided doses (Kimmel et al., 2013). Subsequent studies incorporating more relevant variants showed significantly improved outcomes (Limdi et al., 2015). Determining appropriate CPGx tests to be used for each patient based on relevant gene variants is a potential barrier providers face in implementing PGx testing services.

One of the challenges in determining an appropriate CPGx test is the lack of publicly available information describing them. In a study of direct-to-consumer genetic tests in 2016, less than one-third of tests identified had specific gene variant selection information available (Hall et al., 2017). The National Institutes of Health (NIH) created the Genetic Testing Registry (GTR) in 2010 to collect genetic test information and to enhance their availability, validity, and usefulness (Rubinstein et al., 2013). Information about the tests is voluntarily reported by the commercial clinical laboratories who developed them and is updated regularly. The GTR is considered one of the most valuable genetic testing repositories and is often cited in PGx guidelines.

There are additional resources that support PGx implementation. The Clinical Pharmacogenomics Implementation Consortium (CPIC) helps by creating, curating, and posting freely available, peer-reviewed, evidence-based, updatable, and detailed gene/drug clinical practice guidelines (Relling and Klein, 2011). The Pharmacogenomics Knowledge Base (PharmGKB) is another resource that curates and disseminates knowledge about clinically actionable gene-drug associations, genotype-phenotype relationships, and gene frequency data (Whirl-Carrillo et al., 2012). Finally, specifically for variant selection guidance, the Association for Molecular Pathology (AMP) has established a two-tier evidence-based recommendation system to help laboratory professionals select appropriate gene variants in genotyping assays for CYP2C9 and CYP2C19 (Pratt et al., 2018, 2019). These resources can aid clinicians to know what variants would be appropriate for their patients in CPGx tests.

In our study, we specifically evaluate CPGx tests and their variant selection practices with respect to CYP enzymes. This is due to the critical role CPGx tests and CYP enzymes play in the implementation of preemptive CPGx testing programs. We leverage valuable resources (GTR, CPIC, PharmGKB, and AMP) to perform our evaluation of the current landscape of detection rates of available CPGx tests based on known variant frequencies across various ethnic populations. A list of potential CPGx tests were identified utilizing the GTR, which was subsequently filtered to only include CYP relevant tests. For subsequent analysis, published CPIC guidelines were utilized to identify CYP enzyme phenotypes of interest. These in combination with PharmGKB gene frequency data allowed us to determine the extent genotype predicted phenotypes of interest occur in diverse populations and how well CPGx tests identify them. In our evaluation, gene coverage percentages were determined and the detection rate of CPGx tests were calculated covering five CYP enzymes across various ethnic groups.

## Materials and Methods

### Identification of PGx Tests

The GTR was used to identify PGx tests from 02/23/2021 to 02/26/2021. As keywords, “pharmacogenetic” or “pharmacogenetics” or “pharmacogenomics” or “pharmacogenomic” were used for the search. The resulting PGx test list was filtered based on the following inclusion criteria: assays that used “targeted variant analysis” as their test methodology, CPGx tests including at least two genes, and publicly available gene variant selection information (Supplementary Figure 1). Exclusion criteria for potential CPGx tests included assays using alternative sequencing techniques (whole genome or exome sequencing), assays only including one gene, and CPGx tests without publicly available variant selection information. Gene and variant coverage for the CYP enzymes, specifically, CYP2B6, CYP2D6, CYP2C9, CYP2C19, and CYP3A5, were investigated due to their well-studied gene frequencies and role in PGx practice. Variants considered for analysis of gene selection, gene coverage percentage, and detection rate were those included in PharmGKB gene frequency tables (See Supplementary Table 1 for detailed list).

### Calculation of CPGx Test Coverage Percentage

To illustrate the extent of the number of variants included in PGx tests relative to the number of known variants, a variant coverage percentage was calculated for each CPGx test. Gene coverage percentage is the total number of variants targeted by a CPGx test divided by the total known variants (Eq. 1). Total known variants were the number of listed variants within PharmGKB gene frequency tables.


$$\mathrm{CPGx test coverage percentage}=\frac{\mathrm{Number of variants targeted}}{\mathrm{Total number of known variants}}x\;100$$

Equation 1: Calculation of CPGx test coverage percentage. The number of variants targeted was found by identifying which variants each CPGx test selected from a search using the GTR and other resources. The total number of chosen variants were then summed, in which the total number of known variants was the number of listed variants within the PharmGKB gene frequency tables.

### Calculation of Detection Rate

Diplotype to phenotype translation and diplotype frequencies were obtained from the PharmGKB database for CYP2B6, CYP2D6, CYP2C9, CYP2C19, and CYP3A5. These reported frequencies include nine different ethnic groups. The diplotypes included in PharmGKB lists are based on the Pharmacogene Variation (PharmVar) database and the resulting diplotypes from their listed core variants (Gaedigk et al., 2018). These resources, combined with PGx test information from the GTR, were used to calculate the detection rate of CPGx tests in the different ethnic groups.

Potential diplotypes for CYP enzyme subclasses were filtered to only include those that predict “altered metabolizer” phenotypes. A genotype-predicted phenotype is considered an “altered metabolizer” when it results in a required alteration of medication therapy to dosage or medication choice according to published CPIC guidelines (Birdwell et al., 2015; Hicks et al., 2015; Bell et al., 2017; Hicks et al., 2017; Desta et al., 2019; Theken et al., 2020; Crews et al., 2021; Karnes et al., 2021; Lima et al., 2021). Genotype-predicted phenotypes leading to potential altered metabolizer status are unique to each CYP enzyme sub-group (Table 1). Even though, predicted phenotype does not guarantee adverse drug reactions or suboptimal outcomes, in a pre-emptive approach the resulting predicted phenotypes would influence future therapy decisions. Thus, for our study, we rely on these genotype-predicted phenotypes to define “altered metabolizers.”

**Table 1:** Genotype-predicted phenotypes for each CYP enzyme that cause alteration of medication therapy (altered metabolizing status) based on Clinical Pharmacogenomics Implementation Consortium (CPIC) guidelines.

CYP enzyme	Genotype-predicted phenotypes considered an altered metabolizer status	CPIC guidelines referenced
CYP2B6	Intermediate and poor metabolizers	Efavirenz and efavirenz- containing antiretroviral therapy
CYP2C19	Ultra-rapid, rapid, likely intermediate, intermediate, likely poor, and poor metabolizers	Proton pump inhibitors Selective serotonin reuptake inhibitors Tricyclic antidepressants Clopidogrel
CYP2C9	Activity scores less than 2	Phenytoin Fosphenytoin NSAIDs
CYP2D6	Activity scores less than 1.25 and greater than 2.25	Opioids Selective serotonin reuptake inhibitors Tricyclic antidepressants Atomoxetine Ondansetron Tropisetron
CYP3A5	Intermediate and extensive metabolizers	Tacrolimus

Population altered metabolizer frequency is the sum of all altered metabolizer diplotype frequencies for each ethnic group. Detectable altered metabolizer frequency represents the total frequency of altered metabolizers, where a given CPGx test contains both gene variants within its diplotype (detectable diplotypes). Detection rate is the proportion of individuals with altered metabolizing genotype predicted phenotypes that have detectable diplotypes. Therefore, it is calculated by dividing the detectable altered metabolizer frequency by the population altered metabolizer frequency (Eq. 2). Each ethnic group represented in gene frequency tables has a detection rate value calculated for each CPGx test.


$$\mathrm{Detection Rate}=\frac{\mathrm{Detectable altered metabolizer frequency}\;}{\mathrm{Population altered metabolizer frequency}}x\;100$$

Equation 2: Calculation of detection rate. Altered metabolizer phenotypes are defined as genotype predicted phenotypes resulting in an alteration of medication therapy. Detectable altered metabolizer frequency is the sum of all altered metabolizer diplotypes frequencies, where both gene variants are included in the CPGx test. Population altered metabolizer frequency is the sum of all altered metabolizer diplotypes frequencies.

Association for Molecular Pathology Tier 1 and Tier 2 recommendations were evaluated for CYP2C9 and CYP2C19. Detection rate was calculated for each tier in the same way it was calculated for prospective CPGx tests. These results show the extent these recommended tiers are representative of diverse ethnic groups and allow for comparison of current PGx tests against AMP recommendations. Since only publicly available datasets were used and no animal study was conducted, this study did not require IRB or IACUC approval. All analysis codes are available at https://github.com/sayer108/CPGx_test_evaluation.

## Results

### Genetic Testing Registry Search Results

**Figure 1:**
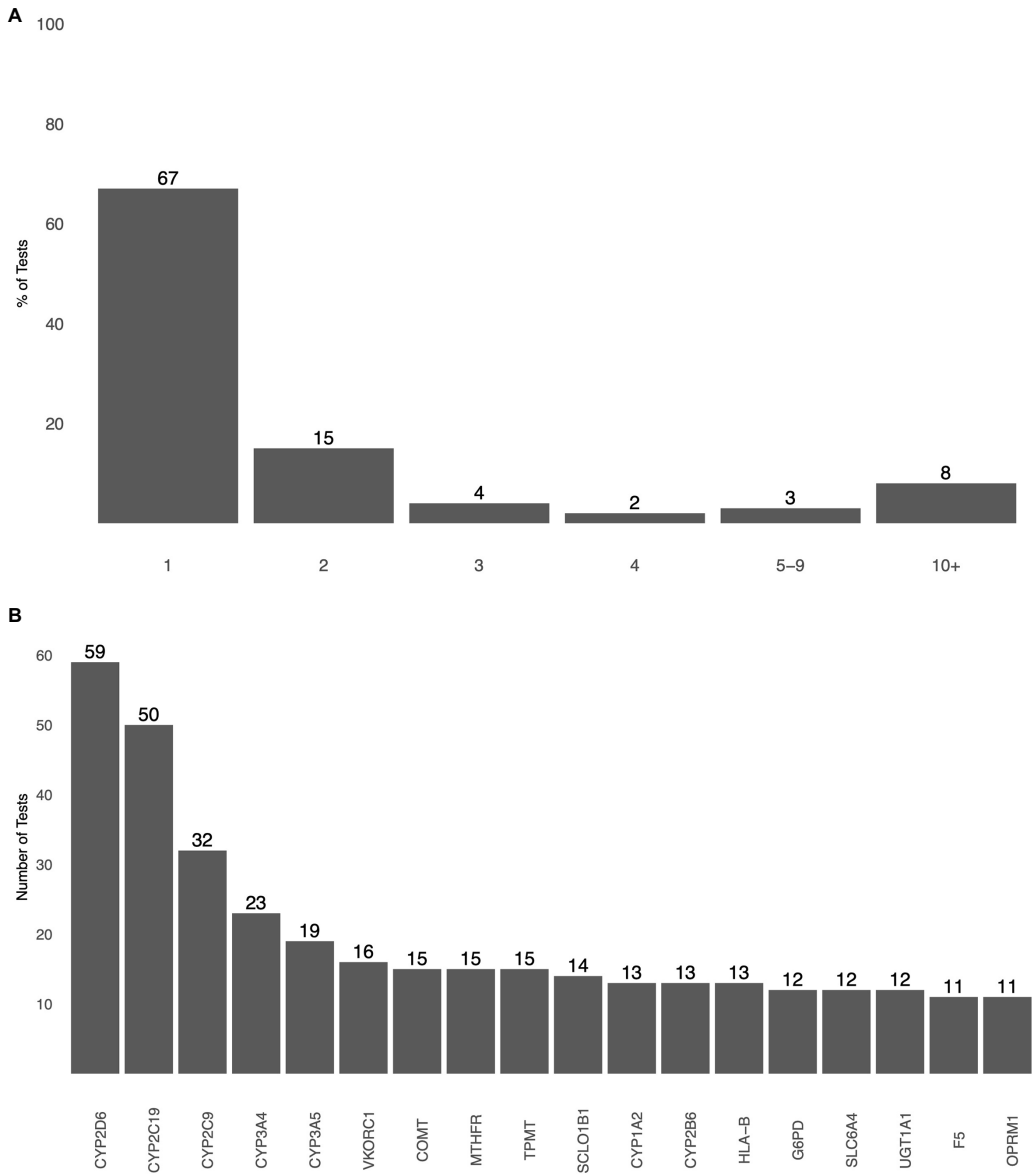
**(A)** The total number of genes selected by pharmacogenomics (PGx) tests as detailed by the test information included in the Genetic Testing Registry (GTR). **(B)** Most commonly selected genes by PGx tests based on gene selection information included in GTR.

A search of the GTR showed 178 potential PGx tests, 56 of them being CPGx tests (Figure 1A). Gene selection information was publicly available for 25% of CPGx tests, only 14 of 56 of them. CYP2C9, CYP2C19, and CYP2D6 were selected by 11 CPGx tests; only six CPGx tests covered CYP2B6. The most commonly selected gene targets in CPGx tests were CYP enzymes, with CYP2D6 and CYP2C19 being selected by 50 different tests (Figure 1B; Diagnostics R, 2021; Genetics C, 2021; Genetics H, 2021; Genomics A, 2021; Health A, 2021a,b; Incorporated CH, 2021; Indiana University School of Medicine DoDG-PL, 2021; Invitae, 2021; Laboratories KD, 2021; Lineagen, 2021; OneOme, 2021; Services PML, 2021).

### Prevalence of Altered Metabolizing CYP Phenotypes

**Figure 2:**
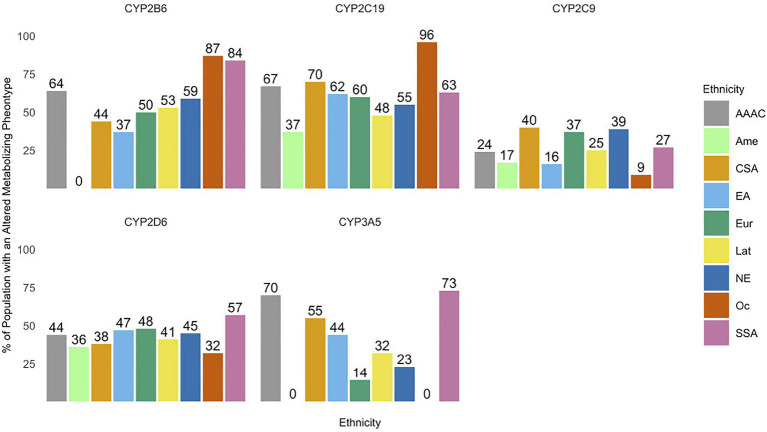
The prevalence of altered metabolizing phenotypes of cytochrome P450 (CYP) enzymes across various ethnic groups. Abbreviations for ethnic groups are as follows: AAAC, African American/Afro-Caribbean; Amer, American; CSA, Central/South Asian; EA, East Asian; Eur, European; Lat, Latino; NE, Near Eastern; Oc, Oceanian; and SSA, Sub-Saharan African. ^*^Values of 0 are due to the lack of variant frequencies documented in the Pharmacogenomics Knowledge Base (PharmGKB) database for a specific ethnic group.

Altered metabolizer genotype predicted phenotypes make up a significant proportion of population for all ethnic groups analyzed within each CYP enzyme subclass (Figure 2). The average altered metabolizer frequencies (range) across ethnic groups for each CYP enzyme subclass were CYP2B6 67% (50–87%), CYP2C19 62% (37–96%), CYP2C9 26% (9–40%), CYP2D6 23% (32–57%), and CYP3A5 44% (14–73%).

### Coverage Percentage and Detection Rate of Pharmacogenomic Tests

The detection rate of PGx tests was calculated for 14 CPGx tests that covered one or more of the following CYPs: CYP2B6, CYP2C9, CYP2C19, CYP2D6, and CYP3A5. The detection rate of CPGx tests varied significantly, with values ranging from 24 to 100%. The average overall detection rate and range of values of detection rate of the CPGx tests for all ethnic groups within each CYP enzyme sub-group were CYP2B6 (77.6%, 43–100%), CYP2C9 (88.4%, 24–100%), CYP2C19 (92.3%, 32–100%), CYP2D6 (81.4, 56–100%), and CYP3A5 (100%, 100–100%; Supplementary Tables 2–8; Supplementary Figure 2).

**Figure 3:**
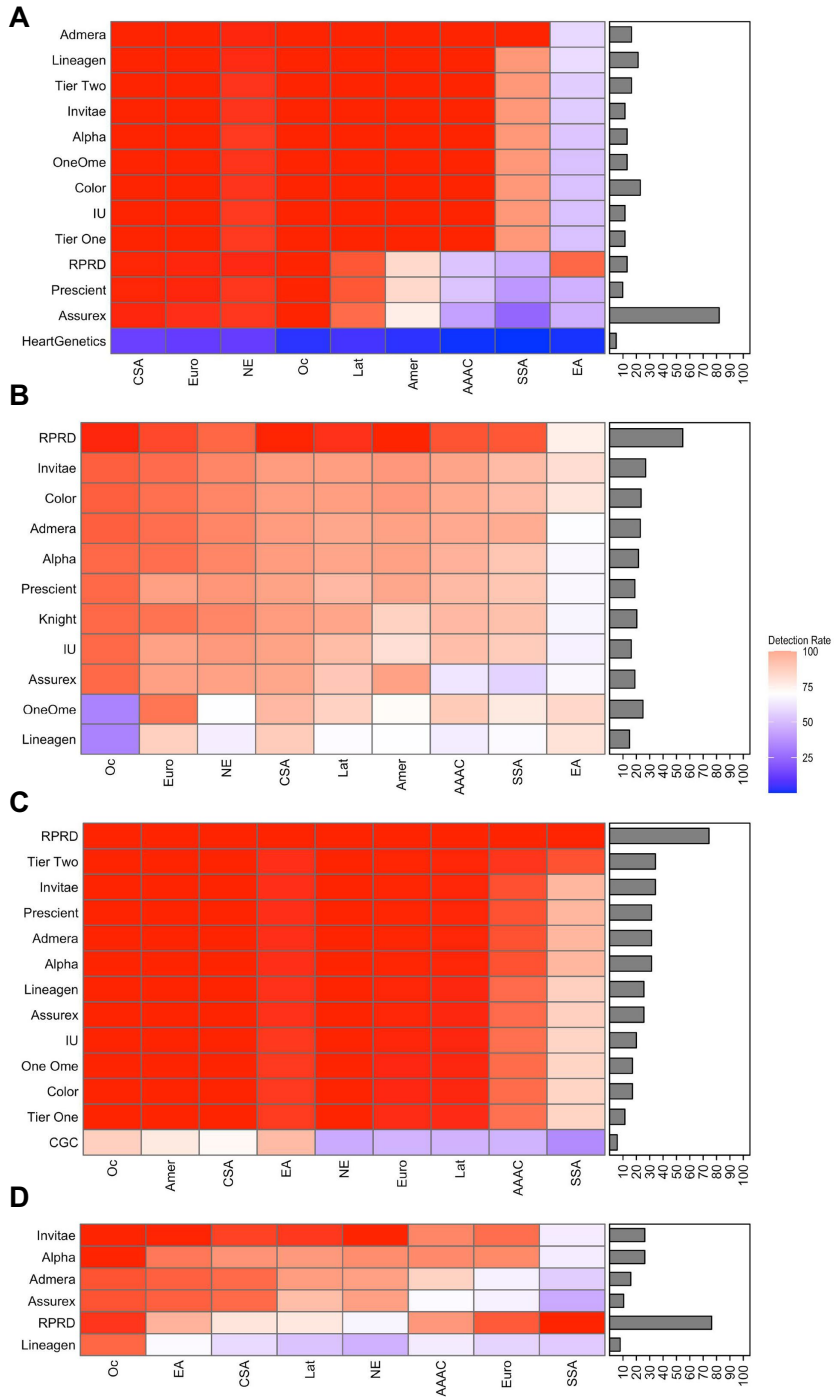
Heatmaps displaying detection rate values and coverage percentage of PGx tests covering **(A)** CYP2C9, **(B)** CYP2D6, **(C)** CYP2C19, and **(D)** CYP2B6. The brighter the red, the higher the detection rate. On the contrary, the darker blue, the lower the detection rate. In addition to the PGx tests, detection rate was calculated for selected Tier 1 and Tier 2 alleles of Association for Molecular Pathology (AMP) recommendations for CYP2C9 and CYP2C19. PGx test coverage percentage is included with each heatmap as a bar chart on the right side of each heatmap. Abbreviations for ethnic groups listed on the bottom of the figure are as follows: Amer, American; CSA, Central/South Asian; EA, East Asian; Eur, European; Lat, Latino; NE, Near Eastern; Oc, Oceanian; and SSA, Sub-Saharan African.

The PharmGKB lists 61 potential variants for CYP2C9; gene coverage percentage ranged from 4.9 to 23% for CPGx tests covering CYP2C9. The detection rate for the East Asian (EA) population was lower than the rest of the ethnic groups, with an average of 55% across CPGx tests. All other ethnic groups had an average detection rate of 70% or higher. AMP Tier 1 and Tier 2 recommendations also had very low detection rates for the EA population, 51 and 55%, respectively. Both tiers had detection rates of 95% or higher for all other ethnic groups (Figure 3A; Supplementary Tables 1, 3, and 8). The highest performing CPGx test among East Asians was the RPRD assay, with a detection rate of 92%. However, for other ethnic groups like Sub-Saharan African (SSA) and African-American Afro-Caribbean (AAAC) populations the CPGx test with the highest detection rate was the Admera assay with a detection rate of 99% (Supplementary Table 8). Similarly, the PharmGKB lists 137 potential variants for CYP2D6 and tests analyzed have gene coverage percentage ranging from 15 to 54%. Average detection rates for all CPGx tests with respect to all ethnic groups ranged from 70 to 90%, with no obvious outliers (Figure 3B; Supplementary Tables 1 and 4). All ethnic groups had the highest detection rate with the RPRD assay except for East Asian populations, where the OneOme assay had the highest detection rate. PharmGKB lists 32 potential variants for CYP2C19 with gene coverage percentages ranging from 5.7 to 75%. The detection rate for the SSA and AAAC populations was lower than the rest of the ethnic groups, 77, and 85%, respectively. The rest of the ethnic groups had average detection rates of 95% or higher for all CPGx tests. RPRD had the highest detection rate for AAAC and SSA populations at 100%. Many of the Ethnic groups had 100% detection rates with several different assays studied. AMP recommended Tier 1 and Tier 2 variants had detection rate values of 95% or higher for all ethnic groups except the SSA and AAAC populations. Tier 1 had a detection rate value of 75% for the SSA population, much lower than Tier 2 at 93% (Figure 3C; Supplementary Tables 1, 5, and 8). The PharmGKB lists 38 potential variants for CYP2B6; gene coverage percentage ranged from 7 to 26.3%. The SSA population had an average detection rate of 63.9% for all CPGx tests, the lowest compared to all ethnic groups. The rest of the groups had average detection rates greater than 70% for all CPGx tests (Figure 3D; Supplementary Tables 1 and 6). The RPRD assay performed best for the SSA population with a detection rate of 100%. Results for CYP3A5 showed a 100% detection rate for all CPGx tests (Supplementary Tables 2, 7, and 8).

## Discussion

In this study, the ranges of detection rates were evaluated for currently available CPGx tests to demonstrate the variability that can occur depending on ethnic background and PGx test selection. Our results showed some ethnic groups clearly have higher and more consistent detection rate scores across CYP enzymes compared to others. This demonstrates that variant selection for the chosen assay can favor some populations more than others. Additionally, even patients of the same ethnic background can receive CPGx tests with drastically different detection rates due to variant selection.

The calculated detection rate of CPGx tests reflected that certain ethnic groups had diminished detection rate values for almost all the CPGx tests analyzed. For instance, if providers seeking to test patients of East Asian descent for CYP2C9 metabolizing status utilized any one of the CPGx tests, the patients metabolizing status would likely be mischaracterized. Additionally, our results show a large variance in PGx test performance, meaning clinicians basing decisions on current test results could be depending on incorrectly characterized phenotypes. The greatest example of this variance was observed with respect to the Oceanian (Oc) population and CYP2D6. While average detection rate was greater than 80% for CPGx tests, there were multiple tests with detection rates between 30 and 40%. If clinicians are aware of the limitations of selective CPGx tests with respect to different ethnic groups and their variability, they can select an alternative; thus, patients will be less likely to experience sub-optimal therapeutic outcomes.

Evaluation of AMP recommended Tier 1 and Tier 2 alleles showed consistent detection rates of 90% or higher across ethnic groups with a few notable exceptions. Specifically, East Asian and SSA populations had significantly lower detection rates for Tier 1 and 2 recommendations for CYP2C9 and CYP2C19, respectively. In these cases, there are a significantly higher number of relevant gene variants compared to the other populations. Despite these exceptions, it was concluded that the results produced support the implementation of standardized gene targets for targeted PGx tests.

Our results demonstrate additional measures that can be taken to further increase the clinical utility of CPGx guided medication therapy. For three out of the five CYP enzyme genes evaluated, peak detection rates were achieved with different CPGx tests depending on ethnic background because of gene selection variations (Supplementary Table 8). Additionally, while higher detection rates were often achieved by tests selecting a higher number of variants, tests selecting fewer variants achieved the same detection rate in many instances. This is due to the fact that low frequency variants contribute minimally to the overall detection rates. These results show providers considering ethnic background in CPGx test selection can ensure their patients’ have the best chance to have their phenotypes correctly identified. Physicians and pharmacists can better identify situations when more selective tests are sufficient and when broader coverage is needed. Careful consideration of CPGx test selection with respect to ethnicity and variant selection has the potential to improve patient care by better charactering altered metabolizing phenotypes. With the growing emphasis on personalized patient care, our results show meaningful ways to further individualize genetic testing and ultimately improve outcomes.

Our results also provide insight into how the clinical utility of informatics approaches can be enhanced. The findings of this study can be potentially implemented in operationalizing PGx test ordering, data sharing, and cascade testing with an integrative informatics approach (Roosan et al., 2021). We demonstrated altered metabolizers are very prevalent in diverse patient populations, and some ethnic populations have a significantly higher proportion of altered metabolizers. This gene variance implies some ethnic populations may be more likely to benefit from the CPGx test. This is consistent with recent data from PGx gene frequency studies in large diverse populations (McInnes et al., 2021). If the patient ethnic background was included in models predicting patients in need of PGx testing, it could improve the models’ performance. Studies have demonstrated ethnicity can be a predictive factor in disease progression and medication efficacy with respect to various cardiovascular diseases and more effective interventions can be initiated when it is considered (Taylor and Wright, 2005). Besides, incorporating ethnic background into the choice of CPGx test can assure more reliable PGx results. Patients in need of PGx tests with expanded gene coverage can be identified, ensuring metabolizing status is assessed correctly. Thus, incorporating factors of ethnicity and ethnicity in informatics tools can improve patient selection for PGx testing and PGx test choice.

This study chose to evaluate CPGx tests due to the emerging evidence showing they are needed to optimize clinical outcomes. Studies comparing CPGx testing against single-gene testing to guide medication decisions for patients with major depressive disorder demonstrated that combinatorial approaches better predicted phenotypes and clinical outcomes than single-gene tests (Winner and Dechairo, 2015). Additionally, CPGx testing approaches provide better opportunities for preemptive PGx practice. In a sample of 10,000 patients preemptively genotyped within the PREDICT program with Vanderbilt University, 91% had at least one actionable variant, and 42% of these patients had been exposed to a risk-associated medication in the past (Hockings et al., 2020). Given the growing body of evidence supporting the clinical benefits of CPGx testing, it is necessary to evaluate these assays.

Utilizing reported racial and ethnic backgrounds in healthcare decision-making is challenging for a variety of reasons. A recent study suggests that ethnicity-based PGx decision-making is limited by intrapopulation genetic variation and fluidity (Goodman and Brett, 2021). Additionally, concerns have been expressed regarding medical decision tools incorporating ethnicity having bias that causes sub-optimal therapeutic outcomes for patients of different ethnic backgrounds (Vyas et al., 2020). However, the FDA and current PGx consortiums have adopted ethnicity-based recommendations for PGx screenings (Chang et al., 2020). This practice has been adopted as a common practice with respect to other disease states. For example, ties between familial genetic history and breast cancer risk have been well documented and this information has been included in decision-making guidelines to screen patients as well (Owens et al., 2019). Until universal PGx testing is widespread, using genetic ancestry can prioritize patients most likely to benefit from a more appropriate CPGx test representing known genetic variations.

There are several limitations to this study. With respect to the availability of data, our evaluation only included voluntarily shared commercial clinical laboratory data, which totaled 14 CPGx tests, 24% of total tests found. This is comparable to an evaluation done on direct-to-consumer genetic tests in 2017, which found only 20% of tests reporting gene selection data (Hall et al., 2017). Therefore, our evaluation is biased by only voluntarily shared data. Gene frequency information is only sufficiently studied/reported in PharmGKB to perform our analysis on CYP enzymes, even though, there are several other genes commonly used in PGx practice.

Including all variants within PharmGKB regardless of their function or frequency in our analysis also has its limitations. For instance, CYP2D6 has nearly 150 variants identified with less than half of them having reported gene frequencies or known metabolic function. While a test may report a very low gene coverage percentage, it may include several common clinically relevant genes to achieve high detection rate. Sound gene selection practices would be reflected in higher detection rate for that test in a specific ethnic population. We considered all known variants reported, rare or frequent, in calculating detection rate. However, detection rates were not considerably influenced by rare variants with unknown or very low frequencies. Diplotype combinations occurring at very low frequencies relative to other genes contribute very little to the total altered metabolizer phenotype frequency in a population. Coverage percentages indicate the extent of known variants included in an assay, while detection rates indicate how well an assay captures the overall diversity of the gene variants in a population. Therefore, both gene coverage percentage and detection rate together provide useful insight into gene variant selection practices for CPGx tests.

Our results highlight the prevalence of false-negative test results when common gene variants are not included in CPGx tests. Recent evaluations of the performance of sequencing technologies utilized in CPGx tests demonstrate they have precision and accuracy values greater than 99.9% (Jablonski et al., 2018). While labs are required to meet standards of analytical performance, they are not evaluated based on the genes they select. Thus, high analytical performance measures can lead to a false sense of security for consumers and providers if relevant genes are not included in the assay. Conflict with respect to false negative test results has come forward with respect to direct-to-consumer genetic tests and breast cancer screenings. Many of the home testing kits incorporate BRCA variants specific to Ashkenazi-Jewish descent, while not including other variants more common in diverse populations leading to false negatives (Landi, 2019). Our data show that this oversight leads to potential false negative results in CPGx tests used in pharmacogenomics practice as well. These false-negatives may significantly impact treatment decisions for patients. Our work supports the continued implementation of institutions like AMP creating recommended genes to include in assays as a standard to ensure reliable results. However, the landscape needs to be continually evaluated as gene frequency and gene functionality information becomes more readily available.

In summary, the proportions of altered metabolizers in a given population can range from 10 to 90% depending on the CYP enzyme sub-class. The majority of assays targeted less than 50% of known gene variants listed in the PharmGKB gene frequency tables. Calculated detection rates of CPGx tests showed high variation across different ethnic groups. Therefore, the patient’s ethnicity receiving the PGx test and the variants targeted by the test should be carefully considered to ensure the optimal utility of PGx.

## Data Availability Statement

The datasets presented in this study can be found in online repositories. The names of the repository/repositories and accession number(s) can be found in the article/Supplementary Material. All analysis codes are available at https://github.com/sayer108/CPGx_test_evaluation.

## Author Contributions

MS, ML, KP, JV, BV, and DP performed research, analyzed the data, and wrote the manuscript. AD and TN performed research, analyzed the data, and reviewed the manuscript. RB and DR analyzed the data and gave critical feedback on the manuscript. MR designed the study, reviewed data analysis, gave critical feedback on manuscript writing, and supervised the research. All authors contributed to the article and approved the submitted version.

## Funding

This study was funded by Chapman University internal grants.

## Conflict of Interest

The authors declare that the research was conducted in the absence of any commercial or financial relationships that could be construed as a potential conflict of interest.

## Publisher’s Note

All claims expressed in this article are solely those of the authors and do not necessarily represent those of their affiliated organizations, or those of the publisher, the editors and the reviewers. Any product that may be evaluated in this article, or claim that may be made by its manufacturer, is not guaranteed or endorsed by the publisher.
