# Supplementary material for: Clinical Implications of Combinatorial Pharmacogenomic Tests Based on Cytochrome P450 Variant Selection
Source: Front Genet. 2021 Sep 28;12:719671. doi: 10.3389/fgene.2021.719671 (PMC8506148; doi:10.3389/fgene.2021.719671)
Supplement: Supplementary file 1 [file Data_Sheet_1.docx]

**Supplementary File:**


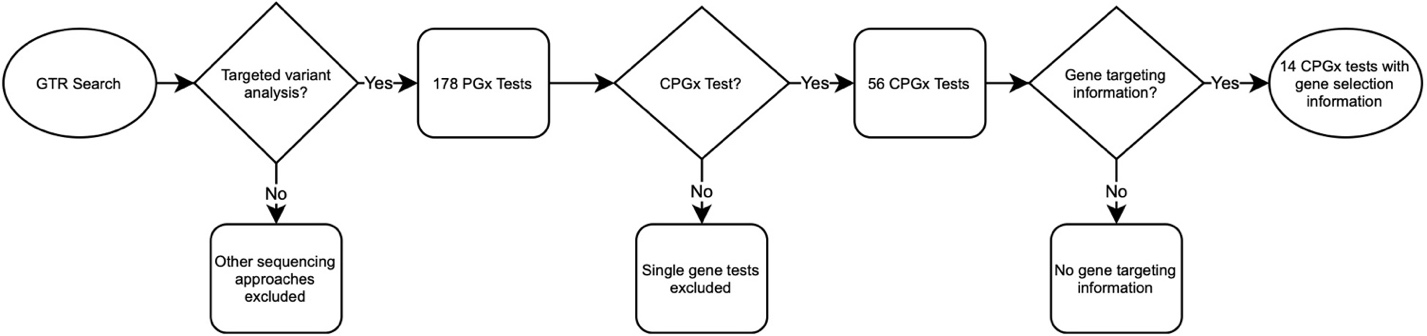


**Supplementary Figure 1:** Overview of combinatorial pharmacogenomics (CPGx) tests selection process from the Genetic Testing Registry (GTR) for analysis. Potential CPGx tests from GTR were eligible for analysis if they used targeted variant analysis, targeted multiple genes, and had variant selection information available.


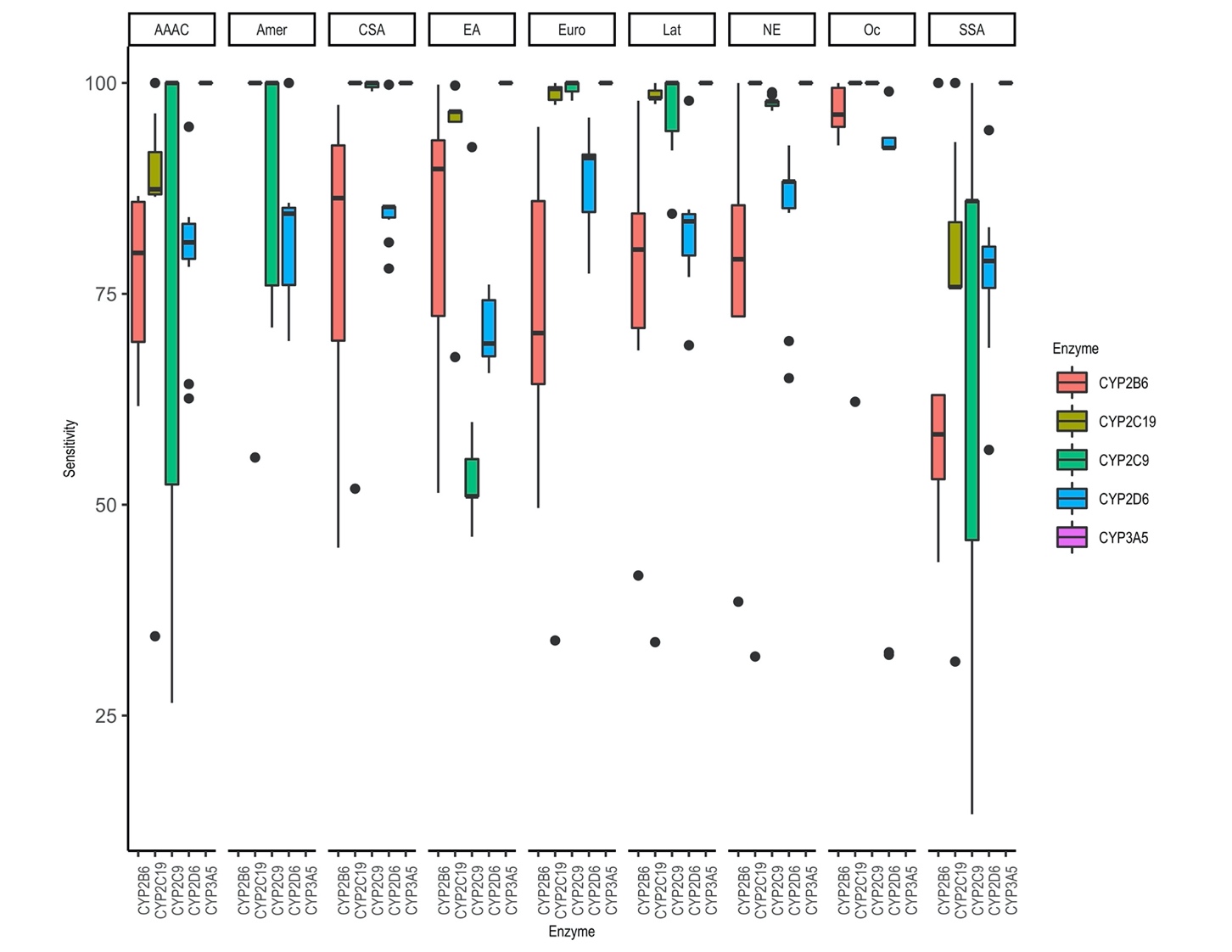


**Supplementary Figure 2:** **Distribution of Combinatorial Pharmacogenomics Test Detection Rate Across Different Ethnic Groups**

Boxplot showing the spread of CPGx detection rates for each ethnic group within each CYP enzyme subclass. Abbreviations for ethnic groups listed on the top of the figure are as follows: Amer (American), CSA (Central/South Asian), EA (East Asian), Euro (European), Lat (Latino), NE (Near Eastern), Oc (Oceanian), and SSA (Sub-Saharan African).

**Supplementary Table 1:** Variants considered for each CYP enzyme isoform for gene coverage percentage and detection rate analysis. All variants listed were those included in the PharmGKB gene frequency tables. Notations such as “x2” or “≥2” indicate gene duplicates.

| **CYP Enzyme** | **Gene Variants** |
| --- | --- |
| CYP2B6 | *1, *2, *3, *4, *5, *6, *7, *8, *9, *10, *11, *12, *13, *14, *15, *17, *18, *19, *20, *21, *22, *23, *24, *25, *26, *27, *28, *29, *30, *31, *32, *33, *34, *35, *36, *37, *38 |
| CYP2C19 | *1, *2, *3, *4, *5, *6, *7, *8, *9, *10, *11, *12, *13, *14, *15, *16, *17, *18, *19, *22, *23, *24, *25, *26, *28, *29, *30, *31, *32, *33, *34, *35, *36, *37, *38 |
| CYP2C9 | *1, *2, *3, *4, *5, *6, *7, *8, *9, *10, *11, *12, *13, *14, *15, *16, *17, *18, *19, *20, *21, *22, *23, *24, *25, *26, *27, *28, *29, *30, *31, *32, *33, *34, *35, *36, *37, *38, *39, *40, *41, *42, *43, *44, *45, *46, *47, *48, *49, *50, *51, *52, *53, *54, *55, *56, *57, *58, *59, *60, *61 |
| CYP2D6 | *1,*1x2,*1≥3, *2, *2x2, *2≥3, *3, *3x2, *4, *4≥2, *5, *6, *6x2, *7, *8, *9, *9x2, *10, *10x2, *11, *12, *13 , *14 , *15, *17, *17x2, *18, *19, *20, *21, *22, *23, *24, *25, *26, *27, *28, *29, *29x2, *30, *31, *32, *33, * 34, *35, *35x2, *36, *36x2, *37, *38, *39, *40, *41, *41x2, *41x3, *42, *43, *43x2, *44, *45, *45x2, *46, *47, *48, *49, *50, *51, *52, *53, *54, *55, *56, *57, *58, *59, *60, *61, *62, *63, *64, *65, *68, *69, *70, *71, *72, *73, *74, *75, *81, *82, *83, *84, *85, *86, *87, *88, *89, *90, *91, *92, *93, *94, *95, *96, *97, *98, *99, *100, *101, *102, *103, *104, *105, *106, *107, *108, *109, *110, *111, *112, *113, *114, *115, *116, *117, *118, *119, *120, *121, *122, *123, *124, *125, *126, *127, *128, *129, *130, *131, *132, *133, *134, *135, *136, *137, *138, *139 |
| CYP3A5 | *1, *2, *3, *4, *5, *6, *7, *8, *9 |

**Supplementary Table 2: Summary Table of Detection Rate of Combinatorial pharmacogenomics (CPGx) Tests** This table shows the average, standard deviation (SD) and range of detection rates of all CPGx tests for each ethnic group within each enzyme subclass. NA values are present due to no gene frequency data being available in the PharmGKB database

Abbreviations for ethnic groups utilized are as follows: EA: East Asian, SSA: Sub-Saharan African, AAAC: African-American/Afro-Caribbean, Euro: European, NE: Near Eastern, Lat: Latino, Oc: Oceanian, CSA: Central/South Asian, Amer: American

**Supplementary Table 3: Coverage Percentage and Detection Rate of Combinatorial pharmacogenomics (CPGx) Tests Covering CYP2C9** The variant number represents the total number of variants targeted by each CPGx test. Subsequent columns are the calculated detection rates for CPGx tests for each ethnic group.

EA: East Asian, SSA: Sub-Saharan African, AAAC: African-American/Afro-Caribbean, Euro: European, NE: Near Eastern, Lat: Latino, Oc: Oceanian, CSA: Central/South Asian, Amer: American

**Supplementary Table 4: Coverage Percentage and Detection Rate of Combinatorial pharmacogenomics (CPGx) Tests Covering CYP2D6** The variant number represents the total number of variants targeted by each CPGx test. Subsequent columns are the calculated detection rates for CPGx tests for each ethnic group.

EA: East Asian, SSA: Sub-Saharan African, AAAC: African-American/Afro-Caribbean, Euro: European, NE: Near Eastern, Lat: Latino, Oc: Oceanian, CSA: Central/South Asian, Amer: American

**Supplementary Table 5: Coverage Percentage and Detection Rate of Combinatorial pharmacogenomics (CPGx) Tests Covering CYP2C19** The variant number represents the total number of variants targeted by each CPGx test. Subsequent columns are the calculated detection rates for CPGx tests for each ethnic group.

EA: East Asian, SSA: Sub-Saharan African, AAAC: African-American/Afro-Caribbean, Euro: European, NE: Near Eastern, Lat: Latino, Oc: Oceanian, CSA: Central/South Asian, Amer: American

**Supplementary Table 6: Coverage Percentage and Detection Rate of Combinatorial pharmacogenomics (CPGx) Tests Covering CYP2B6** The variant number represents the total number of variants targeted by each CPGx test. Subsequent columns are the calculated detection rates for CPGx tests for each ethnic group.

EA: East Asian, SSA: Sub-Saharan African, AAAC: African-American/Afro-Caribbean, Euro: European, NE: Near Eastern, Lat: Latino, Oc: Oceanian, CSA: Central/South Asian

**Supplementary Table 7: Coverage Percentage and Detection Rate of Combinatorial pharmacogenomics (CPGx) Tests Covering CYP3A5** The variant number represents the total number of variants targeted by each CPGx test. Subsequent columns are the calculated detection rates for CPGx tests for each ethnic group.

EA: East Asian, SSA: Sub-Saharan African, AAAC: African-American/Afro-Caribbean, Euro: European, NE: Near Eastern, Lat: Latino, CSA: Central/South Asian

**Supplementary Table 8: Combinatorial pharmacogenomics (CPGx) Tests with Highest Detection Rates Amongst Different Ethnic Groups** CPGx tests with the highest detection rate for each potential gene and ethnicity combination in the study. Below each listed CPGx test the coverage percentage and detection rate are listed between parenthesis. If multiple tests achieve the highest detection rate, all of the tests achieving that score are listed with the range of their coverage percentages. For CYP3A5, all eight tests had detection rates of 100% with coverage percentages ranging from 44- 88%.

|  | **EA** | **SSA** | **AAAC** | **Euro** | **NE** | **Lat** | **Oc** | **CSA** | **Amer** |
| --- | --- | --- | --- | --- | --- | --- | --- | --- | --- |
| **CYP2C9**  **(coverage %, detection rate)** | RPRD  (83, 92) | Admera  (23, 100) | Alpha,  IU,  Lineagen,  OneOme,  Color,  Admera,  Invitae  (11- 23 , 100) | Alpha,  IU,  Lineagen,  OneOme,  Color,  Admera,  Invitae  (11- 23, 100) | Admera  (23, 99) | Alpha,  IU,  Lineagen,  OneOme,  Color,  Admera,  Invitae  (11- 23, 100) | HeartGenetics  Alpha,  IU,  Lineagen,  OneOme,  Color,  Admera,  Invitae,  Prescient,  Assurex,  RPRD  (5- 83, 100) | Alpha,  IU,  Lineagen,  OneOme,  Color,  Admera,  Invitae  (11--23, 100) | Alpha,  IU,  Lineagen,  OneOme,  Color,  Admera,  Invitae  (11- 23, 100) |
| **CYP2D6**  **(coverage %, detection rate)** | OneOme  (25, 76) | RPRD  (55, 94) | RPRD  (55, 94) | RPRD  (55, 95) | RPRD  (55, 92) | RPRD  (55, 97) | RPRD  (55, 99) | RPRD  (55, 99) | RPRD  (55, 100) |
| **CYP2C19**  **(coverage %, detection rate)** | RPRD  (76, 99) | RPRD  (76, 100) | RPRD  (76, 100) | RPRD  (76, 100) | RPRD,  Lineagen,  IU,  OneOme,  Color,  Admera,  Invitae,  Alpha,  Prescient,  Assurex  (17- 76, 100) | RPRD  (76, 100) | RPRD,  Lineagen,  IU,  OneOme,  Color,  Admera,  Invitae,  Alpha,  Prescient,  Assurex  (17-86, 100) | RPRD,  Lineagen,  IU,  OneOme,  Color,  Admera,  Invitae,  Alpha,  Prescient,  Assurex  (17-76, 100) | RPRD,  Lineagen,  IU,  OneOme,  Color,  Admera,  Invitae,  Alpha,  Prescient,  Assurex  (17-76, 100) |
| **CYP2B6**  **(coverage %, detection rate)** | Invitae  (26, 99) | RPRD  (76, 100) | Inviate  (26, 88) | RPRD  (76, 94) | Invitae  (26, 100) | Invitae  (26, 97) | RPRD,  Invitae  (26- 76, 100) | Invitae  (26, 96) | NA |

EA: East Asian, SSA: Sub-Saharan African, AAAC: African-American/Afro-Carribbean, Euro: European, NE: Near Eastern, Lat: Latino, Oc: Oceanian, CSA: Central/South Asian, Amer: American
